# Impact of a Hospital Information System-Integrated Automated Dispensing Cabinet on Medication Use and Safety in a Tertiary Hospital Emergency Department: A Prospective Before-and-After Study

**DOI:** 10.3390/jcm15134908

**Published:** 2026-06-24

**Authors:** Ryang Soon Lim, Woon-Jeong Lee, Hyen Oh La, Yun-Kyoung Song, Kyung Hee Choi

**Affiliations:** 1 College of Pharmacy, The Catholic University of Korea, Gyeonggi-do 14662, Republic of Korea; ryangsoonlim0@gmail.com (R.S.L.); hola@catholic.ac.kr (H.O.L.); 2 Pharmacy Department, Incheon St. Mary’s Hospital, Incheon 21431, Republic of Korea; 3 Department of Emergency Medicine, Incheon St. Mary’s Hospital, Incheon 21431, Republic of Korea; limleeem@catholic.ac.kr; 4 College of Medicine, The Catholic University of Korea, Seoul 06591, Republic of Korea; 5 College of Pharmacy, Gachon University, Incheon 21936, Republic of Korea

**Keywords:** automated dispensing cabinet, emergency department, medication use, medication safety, medication errors

## Abstract

**Background/Objectives**: Emergency departments (EDs) are particularly prone to medication errors because of urgent treatment environments and high decision density. Automated dispensing cabinets (ADCs) integrated with the hospital information system (HIS) may improve medication safety, yet real-world evidence in centralized-pharmacy settings remains limited. This study evaluated the impact of an HIS-integrated ADC on medication use, medication errors, and nurses’ perception of safety in the ED of a Korean tertiary hospital. **Methods**: In this prospective before-and-after study, prescribing patterns, medication storage, and related costs were compared in the two months before (Phase 1; September–October 2019) and after (Phase 2; May–June 2020) ADC installation. Medication errors reported through the hospital safety incident reporting system were analyzed over corresponding 6-month windows from July 2019 to June 2020. Long-term sustainability was assessed using follow-up data collected from October to November 2023 (Phase 3), and all 46 ED nurses completed a 5-point Likert-scale survey on perceived medication safety. **Results**: Daily injectable prescriptions were unchanged (221.1 ± 34.6 vs. 227.7 ± 35.2; *p* = 0.301), whereas returned injectable prescriptions increased (17.9 ± 5.9 vs. 25.1 ± 6.0; *p* < 0.001) and non-injectable prescriptions decreased (163.1 ± 42.2 vs. 140.0 ± 22.7; *p* < 0.001). The number of medication items stored in the ED storage room declined by 95.6%, with a 92.5% reduction in related maintenance cost. Total medication errors decreased from 41 (74.5%) before to 14 (25.5%) after implementation (*p* < 0.001), with the largest reduction in delivery errors (17 [30.9%] to 2 [3.6%]). These improvements were sustained at the three-year follow-up. Nurses reported high overall satisfaction with medication safety (4.27 ± 0.62 on a 5-point Likert scale). **Conclusions**: Implementation of an HIS-integrated ADC in the ED was associated with improved prescription patterns, fewer reported medication errors, and enhanced perceived medication safety. In addition, these improvements were sustained over time, indicating stable and consistent implementation of the ADC system. Nurses also reported improved perceptions of medication safety.

## 1. Introduction

The process of medication use in healthcare institutions is driven by the interaction between healthcare providers, patients, information, and technology, and most medication errors are caused by the complexity of these interactions [1,2]. Especially in emergency departments (EDs), medical staff must make timely and comprehensive care decisions amid insufficient information about the patient’s condition, urgent treatment environments, and various medical situations. Due to the complexity of this environment, EDs are more likely to make medication errors than other departments and are prone to more medication errors than non-medication errors [3,4,5].

Along with other medical automation technologies, automation technology for the safe provision of medications to patients has been continuously developed over the past 20 years. It is being applied at all stages, including prescription, dispensing, distribution, delivery, and administration [1,6]. Recently, equipment with highly advanced automation technology has been linked with hospital information systems, contributing to the development of a digital health environment that improves efficiency and safety throughout the medical system [7,8]. The first automated dispensing technology was introduced in the 1960s. The automated dispensing cabinet (ADC) was first introduced in the United States in the 1980s and is being increasingly used to control the circulation of illicit drugs other than those used by patients, to automate the drug inventory management process, and to reduce drug errors [9,10,11,12,13]. In the Republic of Korea, ADCs have been developed and used since the 2000s, especially in EDs. This is because Korean medical institutions aimed to improve the efficiency and safety issues caused by the operation of the central pharmacy system, which involves multiple stages such as storage, prescription, dispensing, transport, and administration of drugs to reach the treatment site from the pharmacy [13]. ADCs have been digitized and linked with advanced medical information systems, making their functions more accurate and convenient to increase efficiency and safety.

Further research is still needed to comprehensively evaluate the effects of ADC implementation in diverse clinical settings. A previous study reported high user satisfaction with improvements in time efficiency and medication safety, with satisfaction rates of 74.7% and 79.5%, respectively [14]. In addition, a study conducted in a tertiary hospital ED in Australia also demonstrated a 64.7% reduction in medication errors related to drug selection following ADC implementation [4]. Although these findings suggest that ADCs may contribute to safer medication use, evidence from real-world clinical environments remains limited [15]. Therefore, this study aimed to examine the impact of ADC implementation on medication use and safety in the emergency department of a tertiary hospital in Korea.

## 2. Materials and Methods

### 2.1. Study Design and ADC System Implementation

This study was conducted at a tertiary hospital with approximately 900 beds located in Incheon Metropolitan City, the third most populous city in the Republic of Korea. The pharmacy department adopted a central pharmacy system to manage daily prescriptions for patients in all wards and an individual drug prescription-releasing system with, on average, more than 3300 prescriptions and about 13,000 doses per day. Among these, approximately 300 prescriptions and 800 doses were issued daily for ED patients.

**Figure 1:**
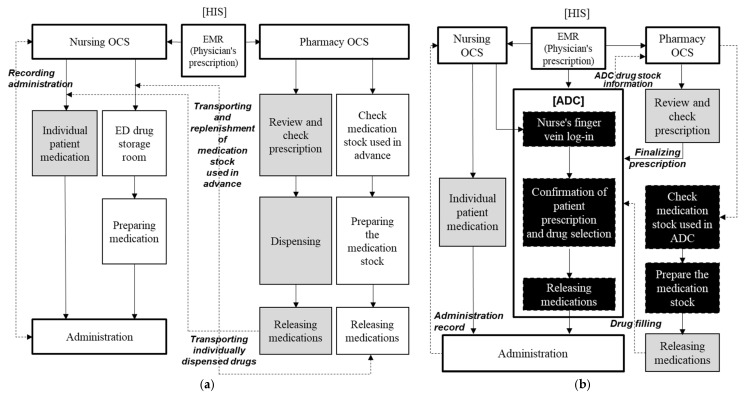
Procedure for using emergency department medication (**a**) before the automated dispensing cabinet (ADC) installation; (**b**) after the ADC installation. Abbreviations: EMR, electronic medical record; HIS, health information system; OCS, order communication system.

The traditional medication supply system from the pharmacy department goes through the following steps: prescriptions are entered into the health information system (HIS), received, and dispensed by the central-pharmacy pharmacist, and the medications are finally delivered to ED nurses. However, if a patient needs medication immediately, the ED stock is used first. In this case, the prescription is reviewed retrospectively in order to replenish the stock. Since no pharmacist is resident in the ED, the medications in the storage room can be used without confirmation from the pharmacist (Figure 1a). The INTIPharm^®^ (JVM, Daegu, Republic of Korea), an ADC consisting of a control station for a supply cabinet, a supply cabinet, and a high-speed dispensing cabinet, was installed in the ED in November 2019. Mainly, frequently used medications are included, while high-risk medications (e.g., narcotics) are excluded. In addition, the minimum stock level for the ADC is determined based on usage and storage volume. The medications not stored in the ADC are supplied via the existing central dispensing system. Whenever a prescription is entered into the electronic medical record (EMR), the details are simultaneously sent to the pharmacy, nursing order communication system (OCS), and ADC. Integration of the ADC with the hospital HIS enables these details to be transferred to the ADC. Only authorized nurses can access the ADC and release medications prescribed for their assigned patients, with radio frequency identification scanning used to ensure the correct medication is outputted. Pharmacists may independently perform clinical reviews of prescriptions entered into the EMR, either before or after nurses access the ADC. In addition, medication usage data from the ADC are reflected in the HIS and pharmacy OCS, enabling the pharmacy team to periodically monitor total usage and re-stock the ADC as necessary (Figure 1b) [16].

### 2.2. Study Periods and Data Collection

Bed utilization rates before and after ADC installation were compared to minimize bias due to changes in the research environment. Reflecting this, we analyzed HIS data to collect prescription and medication consumption data from the central pharmacy in the ED and divided the study into two periods: two months before ADC installation (from September to October 2019, Phase 1) and two months after ADC stabilization (from May to June 2020, as Phase 2). This prospective before-and-after design was intended to evaluate the impact of ADC implementation under real-world clinical conditions. We also collected cases of medication errors according to the medication-use process (prescribing, dispensing, delivery, administration) reported in the hospital safety incident reporting system before (from July 2019 to December 2019) and after ADC installation (from January 2020 to June 2020) [17]. Medication errors were analyzed over a 6-month period to collect sufficient cases and assess temporal trends in safety outcomes. A survey on the impact on practice and medication safety among ED nurses was conducted in September 2020, nine months after full ADC operation began.

Considering the potential influence of the COVID-19 pandemic on ED utilization and workflow, an additional analysis was performed to assess the long-term sustainability of ADC use. Finally, we evaluated the usage status three years after the ADC implementation (October–November 2023, Phase 3) to verify its continued applicability and utilization.

### 2.3. Outcomes

We examined the utilization of ADC by dividing the items and quantities of medications stored in the ADC into injectable and non-injectable forms. Injectable medications applied to ADCs excluded fluids, the product of parenteral nutrient fluids, narcotics, insulin, biological products such as vaccines, and prefilled injections. The following items were examined to determine the impact on medication use before and after ADC installation (Phase 1 and Phase 2):

- Quantity of injectable and non-injectable medications stored in the ADC.
- Number of prescriptions and returned prescriptions for injectable and non-injectable medications.
- Number of injectable medications stored in the ADC in Phase 1 vs. Phase 2.
- Number of medications stored in the ED storage room in Phase 1 vs. Phase 2.
- Number of medication errors and their incident severities before and after the ADC installation.

Incident severity was classified using the National Coordinating Council for Medication Error Reporting and Prevention (NCC MERP) Index for Categorizing Medication Errors, which categorizes events from A to I according to whether an error occurred, whether it reached the patient, and the degree of patient harm. Categories A–D were considered non-harm or administrative/process deviations, whereas Categories E–I were considered clinically significant events associated with actual patient harm, ranging from temporary harm requiring intervention to patient death.

The survey assessing nurses’ perceptions of medication management safety after ADC use was distributed to all 46 nurses working in the ED during the survey period. The questionnaire consisted of 3 questions about the general characteristics of respondents and 4 questions on their perception of medication safety. The survey questions used a 5-point Likert scale (1, strongly disagree; 5, strongly agree).

### 2.4. Statistics

Descriptive analysis was conducted to summarize the data. Categorical variables were presented as frequencies and percentages, and continuous data were expressed as means and standard deviations. Fisher’s exact test or chi-squared test was used to compare categorical data, whereas the independent *t*-test or Mann–Whitney U test was used to compare continuous data. Statistical significance was set at a two-sided *p*-value < 0.05, and all data analyses was performed using IBM SPSS Statistics version 26 for Windows (IBM Corp., Armonk, NY, USA).

Artificial intelligence (AI)-assisted tools were used only for language translation, language editing, and grammar checking during manuscript preparation. Specifically, ChatGPT (GPT-5.5 Thinking, OpenAI) was used to improve the clarity and readability of the English text. The authors reviewed and edited all AI-assisted outputs and take full responsibility for the final content of the manuscript.

## 3. Results

### 3.1. Medication Storage and Utilization in the ADC

Bed utilization rates of the emergency department were similar before and after ADC implementation (Phase 1, 81.0%; Phase 2, 77.9%; *p* = 0.085), indicating comparable clinical conditions. The average number of injectable prescriptions per day in the ED showed no significant difference between Phase 1 and Phase 2 (221.1 ± 34.6 vs. 227.7 ± 35.2, *p* = 0.301), whereas the returned prescriptions for injectable medications increased significantly following ADC implementation (17.9 ± 5.9 vs. 25.1 ± 6.0, *p* < 0.001). However, non-injectable prescriptions significantly decreased from 163.1 ± 42.2 to 140.0 ± 22.7 (*p* < 0.001) (Table 1).

The number of items of emergency medications stored in the ED storage room decreased by 95.6%, from 113 active ingredients in phase 1 to 5 in phase 2, and the total number of medications decreased by 97.8%, from 743 items in phase 1 to 16 in phase 2. The related maintenance cost was also reduced by 92.5% from Korean won (KRW) 2,533,103 in phase 1 to KRW 190,254 in phase 2, showing statistically significant changes (Table 2).

**Figure 2:**
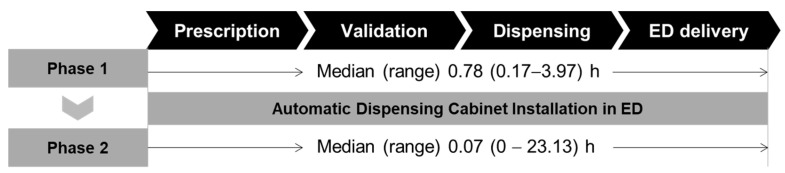
Medication distribution time from prescription to ED delivery before and after automated dispensing cabinet (ADC) installation.

As shown in Figure 2, medication distribution time from prescription in the pharmacy department to ED delivery decreased from a median of 0.78 h before ADC installation to a median of 0.07 h after its installation, representing a 91.0% reduction.

### 3.2. Medication Errors Before and After ADC Implementation

Among the 55 medication errors during the study period, 74.5% occurred before ADC installation, whereas this proportion decreased to 25.5% after implementation (*p* < 0.001). When categorized by the medication-use process, delivery errors showed the greatest reduction, decreasing from 17 cases (30.9%) to 2 cases (3.6%) after ADC implementation. However, the differences in the distribution of medication error types were not statistically significant. When medication errors were further classified according to the NCC MERP severity categories, most events were Category B or C errors. No Category D or E–I events were identified during either study period (Table 3). In addition, high-risk medication-related errors accounted for 14.6% (6/41) of reported medication errors before ADC implementation and 14.3% (2/14) after implementation. Among medication-related errors, errors involving non-ADC medications remained relatively stable (11 vs. 10), whereas those involving ADC-stocked medications decreased from 13 to 2 after ADC implementation.

### 3.3. Nurses’ Perception of Medication Safety After ADC Implementation

All 46 nurses in the ED responded to the survey, yielding a response rate of 100%. The majority of nurses had more than 3 years of clinical experience, and most had 6–12 months of ADC experience. All questions related to safety improvement scored four points or higher as follows: patient medication-related work, 4.37 ± 0.61 points; drug management, 4.30 ± 0.55 points; communication with medical staff, 4.28 ± 0.62 points; and communication with patients (caregivers), 4.11 ± 0.71 points. Nurses’ overall satisfaction with drug safety with ADC use was high at 4.27 ± 0.62 (Table 4).

### 3.4. Long-Term Utilization and Sustainability of ADC Use

**Figure 3:**
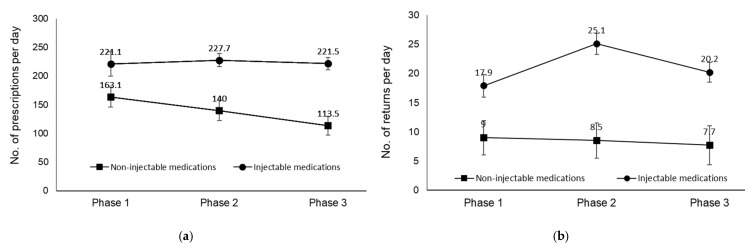
Long-term impact of automated dispensing cabinet (ADC) implementation on (**a**) medication prescription; (**b**) medication returns in an emergency department. Phase 1 represents the pre-implementation period (September–October 2019), phase 2 the immediate post-implementation period (May–June 2020), and phase 3 the follow-up period (October–November 2023).

**Figure 4:**
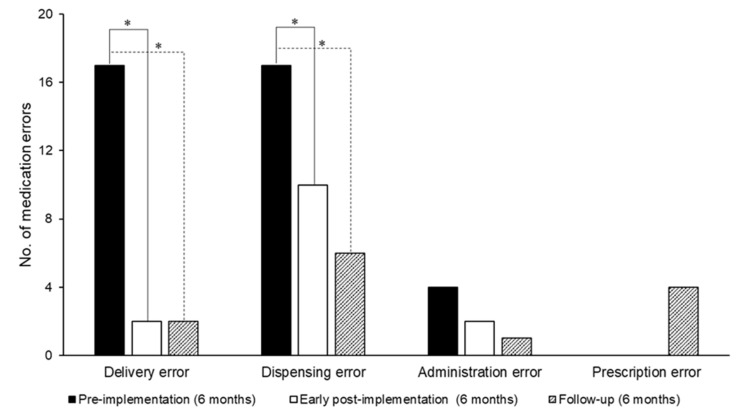
Long-term impact of automated dispensing cabinet (ADC) implementation on medication errors in an emergency department. * means *p* < 0.05.

As shown in Figure 3a, the daily number of prescriptions for injectable medications steadily decreased during the follow-up period. However, the number of returned injectable medications decreased by 20% compared with phase 2 during this period (Figure 3b). As shown in Figure 4, the average number of medication errors remained lower during the follow-up period than before ADC implementation.

## 4. Discussion

The development of digital health systems in conjunction with medical technologies raises expectations for safer and more effective patient care. ADCs integrated with digital hospital systems enhance safety by linking clinical information with automated safety functions. ADCs are being increasingly introduced to reduce errors that can occur during medication use in medical institutions [18,19].

This study is significant for three reasons. First, it confirmed the effect of ADC operation on the safety of medication use in an ED environment different from general wards. Second, it introduced an ADC in a region where the ward pharmacist system is not common and the medication delivery method is centered on the central pharmacy system [20]. Third, it analyzed how the ADC affected the safety of medication use by evaluating changes in prescription, medication use, and errors made in EDs in a general tertiary hospital before and after ADC installation.

Among the medications applied to the ADC, injectable items accounted for the majority, reflecting the need for rapid medication administration in the ED. The use of ADC led to changes in physicians’ prescribing trends. After ADC installation, the number of injectable prescriptions did not change significantly, whereas non-injectable prescriptions significantly decreased. The frequency of injectable returns increased significantly in the early post-implementation period but decreased during the follow-up period. This initial increase in returned injectable prescriptions after ADC implementation may reflect more visible documentation of unused medications through the HIS-integrated ADC workflow [21,22,23]. In addition, medications may have been withdrawn in anticipation of urgent clinical need and subsequently returned when unused, as unnecessary or avoidable ADC withdrawals have been reported in ED settings, where medication orders and treatment plans may change rapidly during transitions such as transfer or discharge [4]. Therefore, the increase in returned injectable prescriptions in the early post-implementation period is plausible, and the subsequent decrease in returns during the follow-up period suggests that ADC-related workflow and medication retrieval practices may have stabilized over time. Overall, ADCs containing frequently used medications may improve medication-use efficiency by enhancing medication access for healthcare providers in the ED [24,25]. Importantly, the sustained patterns observed during follow-up suggest that the impact of ADC implementation was not limited to the immediate post-implementation period.

Regarding the impact of ADCs on medication safety within hospital medication supply systems, it is worth noting that a systematic review study showed that decentralized pharmacy systems, which increase access to medications for medical staff, are more effective in reducing medication errors than central pharmacy systems [26]. ADCs can be considered another form of small, decentralized pharmacy system; therefore, institutions using a central pharmacy system may adopt ADCs as an alternative approach to enhanced medication safety.

Similar to previous studies, ADC use significantly reduced the average monthly number of medication errors in this study (*p* = 0.040). In particular, reductions in dispensing and transport-related errors were observed [2,24,26,27]. Before ADC installation, errors related to incorrect ward delivery were the most common, followed by dosage and medication selection errors, consistent with previous findings [13,28]. After ADC implementation, most errors decreased or did not occur, likely due to controlled access to medications through user authentication and patient-specific dispensing functions.

In light of these favorable outcomes after ADC implementation in the ED, nurses reported high satisfaction with medication safety across all assessed domains, including medication administration tasks, medication management, and communication with healthcare providers and patients. Prior research conducted in hospitals in Qatar and Taiwan has also shown that nurses generally perceive ADCs as improving medication availability, reducing waiting time for medications from the pharmacy, and supporting safer and more efficient medication administration [29,30]. However, qualitative findings from nurses have also identified remaining workflow frustrations, including queuing to access the ADC, incomplete medication availability in the cabinet, delays related to pharmacy verification, stockouts, override-related concerns, and the need for repeated training and workflow adaptation [29,30,31]. A recent scoping review further suggested that ADCs may introduce unintended safety risks, such as medication selection errors when similar drugs are stored together, workarounds, inaccurate inventory information, system-interface delays, and possible over-reliance on technology [32]. Therefore, while our findings suggest that ADC implementation was associated with improved reported safety outcomes and favorable nurse perceptions, the structured survey could not fully capture the underlying reasons for satisfaction, remaining workflow barriers (including screen navigation difficulties, waiting time to access the ADC, verification delays, workarounds during urgent situations, and discrepancies in stock information), or potential unintended risks. Future studies should incorporate qualitative methods, such as focus-group interviews or open-ended survey questions, to better understand healthcare providers’ daily experiences with ADCs and to identify workflow-related safety issues not captured by quantitative satisfaction surveys alone [22,23].

We also found a decrease in human error before and after ADC installation. This result is consistent with a study in 2008 suggesting that about half of medication-related errors are human errors due to lack of attention and that automation is a crucial tool to compensate for such errors [33]. Another study suggests that ADCs improve errors caused by distractions during medication preparation [8,13]. Similar to previous studies, the ADC, which can serve as a medication repository, made medications available in real-time by storing them at a short distance, thereby improving patient care [15,24]. In terms of medication safety management, it was possible to confirm that the correct medications were dispensed through the verification system installed in the device. The ADC contributed to medication safety by establishing a closed-loop medication system with real-time documentation of medication and patient information [19,34,35]. The ADC helped decentralize the medication supply system and led to improved efficiency and accuracy. In addition, since the ADC was digitized and linked with the hospital information system, its functions could be utilized more efficiently [2,8,22].

A strength of this study is that prescription and medication-use data were recorded and extracted from hospital electronic systems, which enhanced data accuracy. Medication errors were also reported anonymously to the hospital’s safety information reporting system, ensuring the report’s objectivity. However, the findings should be interpreted in the context of local implementation of the HIS-integrated ADC interface. The effectiveness of an ADC depends not only on device installation but also on stable interoperability with existing HIS. In particular, the international guidelines recommend that allergy alerts should be reliably transmitted from the HIS to the ADC workflow [22,23]. However, although the ADC used in this study had a real-time stock synchronization function, it did not display allergy information through direct integration with the HIS. Therefore, the scope of future research should also be expanded to include multiple hospitals and diverse ADC configurations incorporated various clinical decision support functions. In addition to medication safety, the benefits of ADC adoption should be evaluated from broader perspectives, including workflow efficiency and economic perspectives.

This study has some limitations. The analysis of errors was limited to those errors reported in the hospital’s safety information reporting system, which may be subject to under-reporting and reporting bias compared to direct observation or chart review to quantify errors [36]. The strength of this system is that any staff member who detects an error can submit a report, rather than only the person directly involved in the error. However, errors that were not detected by others, particularly near misses without patient harm, may have been missed. In addition, ADC implementation may have influenced staff awareness and reporting behavior [14]. Therefore, the results should be interpreted as changes in reported medication errors rather than the true incidence of all medication errors. Nevertheless, the observed reduction in reported medication errors despite this potential increase in reporting would support the robustness of our findings in a conservative direction. In addition, as this study used a before-and-after design and was conducted at a single hospital using a centralized pharmacy system, the findings may not be fully generalizable to other medication distribution systems. Because this study used a single-group, before-and-after design without a parallel control group, such as an ED in a comparable hospital without ADC implementation, the observed changes may have been influenced by confounding variables, secular trends, or other concurrent changes in ED workflow [37]. Although ED bed utilization rates were similar between the study periods, and follow-up data suggested sustained changes, these findings cannot fully exclude the influence of other factors. Therefore, the findings should be interpreted as associations with ADC implementation rather than as evidence of definitive causal effects. In addition, triage level, medication category, and hourly ED patient volume were not available for analysis, which may have resulted in residual confounding. Several aspects of ADC use warrant further consideration, as discussed below.

First, the medications stored in the ED room were not evaluated in this study. Further studies should analyze why medications were excluded from the ADC and how they are used. Although ADCs are devices to improve medication safety and medication management efficiency, system procedures must be followed to achieve this purpose. However, in order to avoid the inconvenience of following system procedures, or at times due to urgent emergency situations, medical staff sometimes do not follow the procedures for generating prescriptions electronically, sending them to ADCs, and receiving medicines from ADCs. In fact, one study reported that the reasons for ADC overrides were often inappropriate [38].

Second, this study evaluated the effectiveness and safety of ADCs at the time immediately after ADC installation, but ongoing evaluation is needed. In this regard, ASHP presents guidelines for the use of ADCs to ensure efficiency and safety in line with the development purpose of ADCs [34,39].

Third, even within a single medical institution, various treatment environments and systems are applied to each clinic department. Therefore, the efficiency and safety of ADCs will reflect various factors depending on the clinic environment. A recent study shows that the time for medication supply and medication shortage problems is reduced in surgical situations [39]. A study reported that the cost of the workload for medication supply in the central pharmacy and clinical departments of tertiary medical institutions was reduced [40].

Finally, another limitation is the potential confounding effect of the COVID-19 pandemic, as part of the post-implementation data collection period overlapped with the early stage of the pandemic in the Republic of Korea. However, the local COVID-19 burden in Incheon, where the study institution is located, was relatively limited during this period, with a total of 341 confirmed cases from January to June 2020, compared with the peak in March 2022 (594,659 cases) [41]. In this study, ED bed utilization rates did not differ significantly between phase 1 and 2, suggesting comparable overall ED utilization. Nevertheless, regional case numbers and bed utilization cannot fully account for possible pandemic-related changes in patient acuity, staffing, workload, medication supply, or reporting behavior. Therefore, residual confounding by the COVID-19 pandemic cannot be completely excluded.

There is a need to study various factors regarding the efficiency and safety of ADCs, reflecting the medical system for medication supply in each country, the medication use system of medical departments within medical institutions, and the work attitude of medical staff. These limitations should be continuously discussed in future studies.

## 5. Conclusions

Implementation of an HIS-integrated ADC in the ED was associated with improved prescription patterns, fewer reported medication errors, and enhanced perceived medication safety. In addition, these improvements were sustained over time, indicating stable and consistent implementation of the ADC system. Nurses also reported improved perceptions of medication safety. However, given the before-and-after study design and potential residual confounding, these findings should be interpreted as associations rather than definitive causal effects.

## Figures and Tables

**Table 1:** Changes in the number of prescriptions per day in the ED between phase 1 and phase 2.

Variables	Prescription Type	Phase 1	Phase 2	*p*-Value
Number of prescriptions per day, mean ± SD	Non-injectable medications (oral, external)	163.1 ± 42.2	140.0 ± 22.7	<0.001
	Injectable medications	221.1 ± 34.6	227.7 ± 35.2	0.301
Number of returned prescriptions per day, mean ± SD	Non-injectable medications (oral, external)	9.0 ± 3.9	8.5 ± 3.6	0.464
	Injectable medications	17.9 ± 5.9	25.1 ± 6.0	<0.001

Abbreviations: SD, standard deviation.

**Table 2:** Changes in emergency medications stored in the ED inventory between phase 1 and phase 2.

Variables	Phase 1	Phase 2	% Change from Phase 1
Number of medication items in the ED storage room	113	5	−95.6%
Number of medications in the ED storage room	743	16	−97.8%
Total cost of emergency medications, KRW	2,533,103	190,254	−92.5%

Abbreviations: KRW, Korean won.

**Table 3:** Changes in medication errors before and after the ADC implementation.

Variables	Before (July–December, 2019), n (%)	After (January–June, 2020), n (%)	*p*-Value
Total number of medication errors	41 (74.5)	14 (25.5)	<0.001
Delivery error	17 (30.9)	2 (3.6)	0.103
Dispensing error	17 (30.9)	10 (18.2)	0.068
• Dose error	4 (7.3)	0	
• Drug selection error	3 (5.5)	5 (9.1)	
• Double dispensing	1 (1.8)	2 (3.6)	
• Others	9 (16.4)	3 (5.5)	
Administration error	4 (7.3)	2 (3.6)	0.638
• Recording error	0	2 (3.6)	
• Dose error	3 (5.5)	0	
• Others	1 (1.8)	0	
Prescription error	0	0	N/A
Others	3 (5.5)	0	0.562
Classification by NCC MERP severity category ^†^			
B—error did not reach the patient	36 (65.5)	14 (25.5)	
C—error reached the patient, no harm	5 (9.1)	0	
D—reached the patient; intervention to preclude harm	0	0	
E–I—error contributed to/resulted in patient harm	0	0	

Abbreviations: ADC, automated dispensing cabinet; N/A, not applicable; NCC MERP, National Coordinating Council for Medication Error Reporting and Prevention. ^†^ Severity assigned from incident narratives, detection stage, and reported harm (provisional first-pass; pending independent dual-reviewer confirmation). Categories B–D = no harm, E–I = harm. Percentages are of the 55 total errors.

**Table 4:** Nurses’ perception of medication safety (5-point Likert scale, *n* = 46).

Variables	Response Score, Mean ± SD
Overall satisfaction	4.27 ± 0.62
Medication administration tasks for patients could be performed more safely. (e.g., timely administration, administration of the correct dose, prevention of medication errors.)	4.37 ± 0.61
Medication use and management tasks in the department could be performed more safely. (e.g., appropriate storage, prevention of medication preparation errors, prevention of breakage, prevention of loss, etc.)	4.30 ± 0.55
Communication among healthcare professionals (physicians, nurses, pharmacists, etc.) was conducted more safely. (e.g., reduction in errors occurring during prescription inquiries, dispensing inquiries, etc.)	4.28 ± 0.62
Communication with patients and caregivers was conducted more safely. (e.g., confirmation of prescribed medications, use of labels, etc.)	4.11 ± 0.71

## Data Availability

The data presented in this study are available upon request from the corresponding author.

## Abbreviations

The following abbreviations are used in this manuscript:

ADC	Automated dispensing cabinet
ED	Emergency department
HIS	Health information system
EMR	Electronic medical record
